# A matched case-control study to assess the association between non-steroidal anti-inflammatory drug use and thrombotic microangiopathy

**DOI:** 10.1371/journal.pone.0202801

**Published:** 2018-08-24

**Authors:** Aiden R. Liu, Ainslie M. Hildebrand, Stephanie Dixon, Jessica M. Sontrop, William F. Clark, Alejandro Lazo-Langner, Danielle Nash, Amit X. Garg

**Affiliations:** 1 Department of Epidemiology & Biostatistics, Western University, London, Ontario, Canada; 2 Institute for Clinical Evaluative Sciences, Ontario, Canada; 3 Division of Nephrology, Department of Medicine, University of Alberta, Edmonton, Alberta, Canada; 4 Division of Nephrology, Department of Medicine, Western University, London, Ontario, Canada; 5 Division of Hematology, Department of Medicine, Western University, London, Ontario, Canada; Istituto Di Ricerche Farmacologiche Mario Negri, ITALY

## Abstract

Several case reports suggest that non-steroidal anti-inflammatory drug (NSAID) use is associated with development of thrombotic microangiopathy (TMA). We conducted a matched population-based case-control study using linked administrative healthcare data in Ontario, Canada to assess the relationship between TMA hospitalization and recent exposure to prescription NSAIDs versus acetaminophen (acetaminophen was chosen as the referent drug because it has no known association with TMA). Cases and controls were drawn from a source population of adults who filled a prescription for either NSAIDs or acetaminophen between 1991 and 2015 (restricted to adults with prescription drug benefits [n = 3.6 million]). We identified 44 eligible cases with a hospital admission for incident TMA and a recent prescription for NSAIDs or acetaminophen. We successfully matched 38 cases (1:4) to 152 controls without TMA on demographics, risk factors for TMA, and indications for NSAID use. Cases and controls were similar with respect to age (71 years) and sex (63% women); however, on average, cases had more comorbidities than controls (12 vs. 14; p<0.05) and more primary care visits in the year before the index date (19 vs. 12; p<0.05). Cases were significantly less likely than controls to have received a recent prescription for NSAIDs (19/38 [50%] vs. 115/152 [76%], respectively; adjusted odds ratio 0.37, 95% confidence interval: 0.16 to 0.84). Results were similar in several sensitivity analyses. Overall, the results of this study do not support a harmful association between NSAID use and the development of TMA.

## Introduction

Thrombotic microangiopathy (TMA) is a pathological process characterized by microangiopathic hemolytic anemia, thrombocytopenia, and organ injury from endothelial damage and systemic microvascular thrombosis. TMA can result from congenital predisposing conditions, or may be acquired through a variety of mechanisms including autoimmunity, complement dysregulation, enteric infection with Shiga toxin, or drug-mediated reactions.[1] TMA may also be a manifestation of several other underlying disorders. The estimated incidence of TMA is 3 to 10 cases per 1,000,000 persons each year.[2]

Several drugs have been implicated as causes of TMA, through either a toxic-mediated response with direct tissue injury, or an immune-mediated response due to drug-dependent antibodies.[3] In 2015, a review article of case studies cited non-steroidal anti-inflammatory drugs (NSAIDs) as a suspected cause of TMA.[3] This is concerning given that NSAIDs are used liberally to treat pain, fever, and inflammation in many common conditions. We performed a comprehensive review of the literature to evaluate the strength of evidence on the possible association between TMA and NSAID exposure. We first searched the bibliographic databases Pubmed, EMBASE, Google Scholar, and Web of Science, and identified 8 case reports suggesting a possible link between TMA and NSAID usage.[4–11] The case reports were critically appraised based on an existing framework adapted from Al-Nouri et al. in order to scrutinize the likelihood of association.[3] A summary of our review is provided in supporting information S1 Table, which includes an assessment of methodological quality and the likelihood that NSAIDs cause TMA. These case reports suggest that an association between NSAIDs and TMA is plausible, but conclusions are limited by inability to elicit cause and effect relationships, several forms of bias, and poor generalizability.[12] We also searched the European Database and Suspected Adverse Drug Reaction Reports[13] (which records reports from the European Economic Areas) and identified 54 cases of TMA with an NSAID cited as a suspected cause. Ibuprofen accounted for the majority of these cases (40), followed by diclofenac (12) and naproxen (2). However, stronger evidence is required to establish an association between NSAIDs and TMA. This prompted us to conduct a population-based case-control study to examine the association between NSAID use and TMA.

## Methods

### Study design

We conducted a population-based case-control study using administrative health care data in Ontario. Ontario is the largest province in Canada, with a population of nearly 14 million people.[14] All Ontario residents have universal access to health care services. In addition, prescription drug coverage is provided under the Ontario Drug Benefit Program to residents over the age of 65, residents of long-term care facilities, and those who receive social assistance or who have high drug costs relative to income. This study was conducted at the Institute for Clinical Evaluative Sciences, which securely holds records from multiple linked databases; these databases record demographics, vital status, and detailed diagnostic and procedural information for all residents of Ontario. We conducted this study according to a pre-specified protocol approved by the Research Ethics Board located at the Sunnybrook Health Centre. All patient data was fully anonymized prior to access and analysis. The reporting of this paper follows the Reporting of studies Conducted using Observational Routinely-collected Data (RECORD) statement (supporting information S2 Table).

### Data sources

This study was performed using data contained in four linked datasets: (1) the Ontario Registered Persons Database, which contains demographic information for all residents of Ontario, (2) the Ontario Drug Benefit database, which contains information on prescription drug use in a subset of the population, (3) the Canadian Institute for Health Information Discharge Abstract Database, which contains information on all hospital admission diagnoses in Ontario, and (4) the Ontario Health Insurance Plan database, which records billings for physician services (including plasma exchange therapy). These datasets were linked using unique encoded identifiers and analyzed at the Institute for Clinical Evaluative Sciences. Approximately 95% of Ontario physicians operate under the fee-for-service payment structure of the Ontario Health Insurance Plan, and the sensitivity and positive predictive value of procedure codes such as plasma exchange is expected to be high as shown with other service payments.[15–17] We defined baseline characteristics and hospital admission diagnoses using the Canadian Institute for Health Information Discharge Abstract Database and the Ontario Health Insurance Plan database.

### Study population

Cases and controls were drawn from a source population of patients who had at least one prescription for an NSAID or acetaminophen dispensed through the Ontario Drug Benefit Program between July 1991 and March 2015. These data were then linked to the Registered Persons Database, and a small proportion of patients with invalid or missing values for age, sex, or health card number (patient identifying number) were excluded. For the purpose of this study, patients prescribed an NSAID were classified as “exposed”, while patients prescribed acetaminophen were classified as “unexposed”. Patients with evidence of both an NSAID and acetaminophen were excluded from the analysis so that we could compare mutually exclusive groups.

Next, we identified all hospitalizations with TMA that fell within a specified window relative to drug initiation through linkage to the Canadian Institute for Health Information–Discharge Abstract Database. The window of time in which we ascertained TMA hospitalization was defined by 1.5 times the variable “day supply” (indicated in the Ontario Drug Benefit database) after prescription start date. For example, if a patient received a 30-day supply of NSAID or acetaminophen, we would look to see if they had record of hospitalization for TMA within 45 days (1.5 x 30 days) from the prescription start date. In cases, the date of first hospitalization for TMA served as an index date. Index dates fell between July 1, 1996 and March 31, 2015 to allow 5 years look back window to describe patients at baseline. Since the remaining patients (controls) were not diagnosed with TMA, we sampled the distribution of index dates from the case population and randomly assigned index dates to controls based on the same distribution of index dates as cases. As we were only interested in incident cases of TMA, we excluded patients who had plasma exchange treatments or a diagnosis of TMA prior to the date of their TMA hospitalization. The remaining patients included (1) cases with a hospital admission code for TMA reflecting new onset TMA (codes defined in supporting information S3 Table), and (2) potential controls that did not have a history of or hospital admission code for TMA.

Given the way we constructed the study sample to efficiently pull data from our data sources, it was expected we would have a substantial number of patients with no evidence of an NSAID or acetaminophen dispensed just prior to the index date (i.e. they had an NSAID or acetaminophen filled between July 1996 and March 2015, but this was well before or after their index date); such patients were excluded from analysis.

Baseline characteristics were assessed using ICD-9 and ICD-10 codes within the five-year period before the index date, with the exception of primary care physician visits, which were assessed in the year before the index date (but not in the 30-day period before the index date to avoid physician encounters possibly related to the TMA; database codes used to define characteristics are presented in supporting information S3 Table). Baseline outpatient drug use was ascertained in the 120-day period before the index date (in Ontario, the maximum supply for a dispensed drug is 100 days).

### Statistical analysis

We matched 4 controls per case based on the following characteristics: age (± 2 years), sex, index date (<6 months), rural residence (population less than 10,000), neighborhood income quintile, and conditions and drugs associated with TMA: malignant hypertension, systemic lupus erythematosus, HIV, sepsis, and use of quetiapine, tacrolimus, sirolimus, cyclosporine, clopidogrel, and ticlopidine. We compared baseline characteristics between the cases and controls using generalized estimating equations, which accounted for the matched design.[18] A p-value of <0.05 was considered to be statistically significant.[19] We used conditional logistic regression to estimate odds ratios (ORs) and 95% confidence intervals (CI) for the association between NSAID use and TMA. We adjusted for any unbalanced baseline characteristics in our models. All analyses were conducted with SAS version 9.3 (SAS Institute Inc.). The authors A. Liu and S. Dixon had access to all aforementioned datasets and performed all data analyses. To comply with privacy regulations for minimizing the chance of patient identification, cells with 1 to 5 patients were reported as ≤5.

To test the robustness of the results, we repeated the analysis twice with either hydromorphone or angiotensin converting enzyme inhibitors (ACE-I) as the referent (unexposed) group. The former was a sensitivity analysis to confirm the results of the primary analysis as hydromorphone is not known to be associated with TMA, and the latter was done to reduce concerns about over-the-counter NSAID use in the control group (as NSAIDs are often avoided in the setting of ACE-I use).

## Results

**Fig 1:**
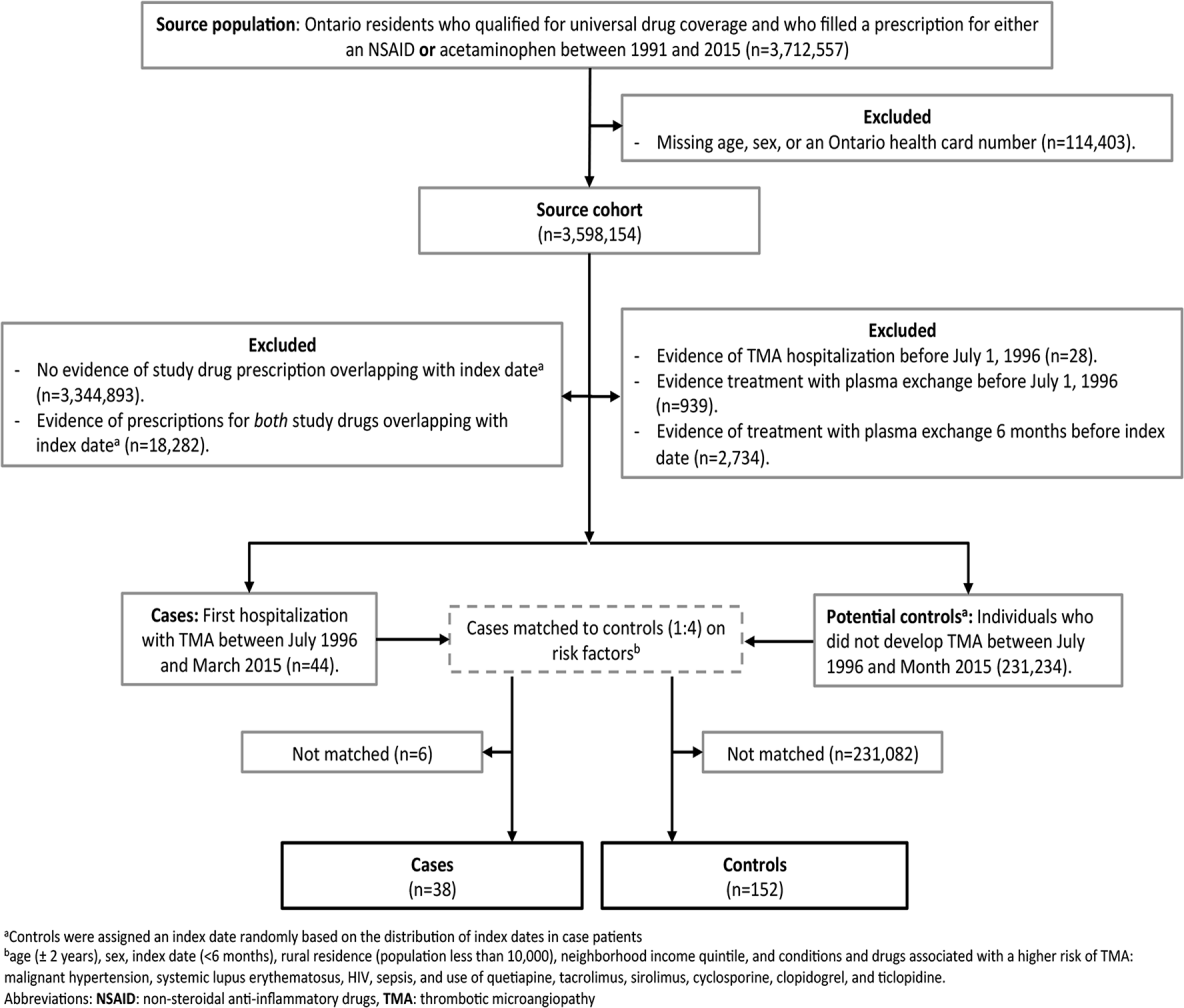
Flow diagram of patient selection with acetaminophen as the referent group.

Patient selection is summarized in Fig 1. We identified 44 eligible cases with a hospital admission for TMA between July 1996 and March 2015. We successfully matched 38 of these cases to 152 controls without TMA. All patients were prescribed either an NSAID or acetaminophen prior to their index date. Hospital admissions for the 38 cases occurred in 28 unique hospitals in Ontario. Within 90 days of their hospital admission, 16/38 cases (42%) received at least one treatment with plasma exchange, 6/38 (16%) received at least one treatment with dialysis, ≤5/38 (≤13%) were admitted to an intensive care unit, and ≤5/38 (≤13%) died. Patients treated with plasma exchange in Ontario for TMA with a recent history of NSAID use had medical chart data abstracted for another study; in this analysis, the diagnostic codes for TMA identified a group of patients (n = 22) with the following median, (25^th^, 75^th^ percentile) blood results as nadir or peak values during hospital stay: hemoglobin 98 (71, 108) g/L, platelets 49 (22, 87) x 10^9^/L, creatinine 180 (87–393) μmol/L and lactate dehydrogenase 1219 (439, 1981) U/L.

Baseline characteristics for the matched sample of cases and controls are presented in Table 1. As expected, demographic characteristics were similar between groups; the mean age was 67 years in each group, 63% were women, 21% lived in a rural area, and 18% were in the lowest income quintile. On average, however, cases had more primary care physician visits than controls (19 vs. 12; p<0.05) and more comorbidities (14 vs. 12; p<0.05). Medications associated with thrombotic microangiopathy (quetiapine, tacrolimus, sirolimus, cyclosporine, clopidogrel, and ticlopidine) were all ≤5 in cases and controls (data not shown).

**Table 1:** Baseline characteristics for patients prescribed NSAIDs or acetaminophen with and without thrombotic microangiopathy (cases and controls, respectively).

	Controls (n = 152)	Cases (n = 38)	P-value^1^
**Demographics**^2^			
Age, no. (%)			
Median (IQR)	71 (65–79)	71 (61–78)	
Mean ± SD	67 ± 16.11	67 ± 16.75	0.47
16–34	9 (5.9%)	≤5	0.23
35–44	8 (5.3%)	≤5	
45–54	7 (4.6%)	≤5	
55–64	11 (7.2%)	≤5	
65–74	61 (40.1%)	15 (39.5%)	
75–84	45 (29.6%)	9 (23.7%)	
≥ 85	11 (7.2%)	≤5	
Women, no. (%)	96 (63.2%)	24 (63.2%)	1.0
Rural residence^3^, no. (%)	32 (21.1%)	8 (21.1%)	1.0
Socioeconomic status^2^, no. (%)			
Quintile 1^4^	28 (18.4%)	7 (18.4%)	1.0
Quintile 2	52 (34.2%)	13 (34.2%)	1.0
Quintile 3 + 4	40 (26.4%)	10	1.0
Quintile 5	32 (21.1%)	8 (21.1%)	1.0
Primary care physician visits, no. (%)^2^			
Median (IQR)	9 (5–15)	14 (7–23)	
Mean ± SD	12 ± 11.98	19 ± 17.06	<0.05
0–2	16 (10.5%)	≤5	<0.05
3–4	20 (13.2%)	≤5	
5–6	24 (15.8%)	≤5	
7–8	14 (9.2%)	≤5	
9–10	15 (9.9%)	≤5	<0.05
≥ 11	63 (41.4%)	23 (60.5%)	
**Comorbidities, no. (%)**^2^			
John Hopkins Aggregated Diagnosis Group Score, no. (%)			
Median (IQR)	12 (9–15)	14 (12–16)	
Mean ± SD	12 ± 3.77	14 ± 3.43	<0.05
≤ 9	44 (28.9%)	10 (26.3%)	<0.05
10–12	41 (27%)		
13–15	42 (27.6%)	14 (36.8%)	
≥ 16	25 (16.4%)	14 (36.8%)	
Malignant hypertension	≤5	≤5	-
Systemic lupus erythematosus	≤5	≤5	-
Cancer	Suppressed	≤5	1.0
Renal transplant	≤5	≤5	-
Osteoarthritis	12 (7.9)	≤5	0.59
Rheumatoid arthritis	12 (7.9)	≤5	0.56
HIV	≤5	≤5	1.0
Sepsis	≤5	≤5	1.0

no.: Number, IQR: interquartile range, SD: Standard Deviation, NSAIDs: Non-steroidal anti-inflammatory drugs.

^1^ P-values are calculated using generalized estimating equations to account for the non-independent correlation structure.

^2^Cells are combined or suppressed to avoid reporting numbers ≤5

^3^Rural residence is defined as population < 10,000

^4^Quntiles are ranked from lowest to highest (i.e. Quintile 1 = lowest, Quintile 5 = highest).

As shown in Table 2, cases were significantly less likely than controls to have received a recent prescription for NSAIDs (19/38 [50%] vs. 115/152 [76%], respectively; OR 0.32, 95% CI: 0.15–0.68). Results remained the same after adjusting for baseline comorbidity scores and number of primary care visits (adjusted OR 0.37, 95% CI: 0.16–0.84).

**Table 2:** The association between NSAID use and thrombotic microangiopathy, with acetaminophen as a reference group. Odds ratios derived from a conditional logistic regression model^1^.

	Cases of TMA (n = 38)	Controls (n = 152)	Odds Ratio (95% confidence interval)	
			Unadjusted	Adjusted^2^
Acetaminophen	19 (50%)	37 (24%)	1.0 (referent)	1.0 (referent)
NSAIDs^3^	19 (50%)	115 (76%)	0.32 (0.15–0.69)	0.37 (0.16–0.84)

^1^Cases were matched to controls in a 1:4 ratio based on age (± 2 years), sex, index date (<6 months), rural residence (population less than 10,000), neighborhood income quintile, and conditions and drugs associated with thrombotic microangiopathy: malignant hypertension, systemic lupus erythematosus, human immunodeficiency virus, sepsis, and use of quetiapine, tacrolimus, sirolimus, cyclosporine, clopidogrel, and ticlopidine.

^2^Adjusted analysis included the variables John Hopkin’s Aggregated Diagnosis Group score and primary care physician visits.

^3^NSAIDs: Non-steroidal anti-inflammatory drugs.

Results were similar in several sensitivity analyses. There was no association between NSAID use and TMA when the referent group was hydromorphone (small cell counts prevent us from presenting the data). Similarly, there was no association between NSAID use and TMA when the referent group was ACE-I (OR 0.82, 95% CI: 0.45–1.49) (selection, baseline characteristics and outcomes are presented in supporting information S1 Fig, S4 and S5 Tables, respectively). As well, we further examined 8 exposed cases who received a second prescription for an NSAID in the year following the TMA hospital discharge date. None of these patients had a re-hospitalization with TMA in the 30 days after the second NSAID prescription.

## Discussion

There are over 30 million daily users of NSAIDs worldwide; many patients who present with TMA will have a prior history of NSAID use.[20,21] Previous case reports suggest NSAIDs could be associated with TMA.[4–8] We conducted this case control study to better understand this possible association. We found that prescription NSAID use was not associated with a higher risk of TMA when compared with prescription acetaminophen and other drugs not known to cause TMA. These results suggest that TMA should not be attributed to a history of NSAID use.

Our findings prompted us to re-examine the case reports.[3–11] The most common reason the reports suggested NSAIDs as the cause of TMA was simply because there was no other identified cause present. Furthermore, no research to date provides a strong biological basis for a higher risk of TMA with NSAIDs. This would suggest that, at the very least, NSAIDs are not associated with a higher risk of TMA, which is consistent with our findings.

None of the case reports addressed the topic of re-introduction of an NSAID after an episode of TMA. It would be concerning if NSAID use after a TMA episode resulted in TMA reoccurrence. In our study, we found that 8 (42.1%) exposed cases received a repeat prescription for NSAIDs in the year following their TMA-associated discharge date. No patient was re-hospitalized with TMA in the 30 days after the follow-up NSAID prescription. Thus, these data do not support avoiding NSAID use in patients with a prior history of TMA.

To our knowledge, this is the first cohort study to explore any drug-induced cause for TMA. Since TMA is a rare event (< 1 per 100,000)[1,22,23], our use of large healthcare databases was opportune, as we captured all TMA cases in our population of interest for the entire province of Ontario over two decades. However, as with all observational studies, our results are subject to residual confounding. Despite controlling for many well-known risk factors for TMA and important indications of NSAID use, not all the characteristics are coded perfectly in our data sources, which were collected for the primary purpose of healthcare administration rather than research.

While we included patients of all ages, the majority of information gathered from the Ontario Drug Benefit database was limited to patients beyond the age of 65. This was apparent in the mean age of 67 years for our patients. Future research focusing on a younger population could expand on our findings.

In conclusion, the results of this study do not support NSAIDS as a cause of TMA.

## Supporting information

S1 Fig Flow diagram of patient selection with angiotensin-converting enzyme inhibitors as referent group. (DOCX)

S1 Table Case report appraisal. (DOCX)

S2 Table The RECORD statement–checklist of items, extended from the STROBE statement, that should be reported in observational studies using routinely collected health data. (DOCX)

S3 Table Coding definitions for cohort build and baseline characteristics. (DOCX)

S4 Table Baseline characteristics for patients prescribed NSAIDs^1^ or ACE-inhibitors^1^ with and without thrombotic microangiopathy (cases and controls, respectively). (DOCX)

S5 Table The association between NSAID use and thrombotic microangiopathy, with ACE inhibitors as a reference group. Odds ratios derived from a conditional logistic regression model.
(DOCX)
