# Supplementary material for: A matched case-control study to assess the association between non-steroidal anti-inflammatory drug use and thrombotic microangiopathy
Source: PLoS One. 2018 Aug 24;13(8):e0202801. doi: 10.1371/journal.pone.0202801 (PMC6108507; doi:10.1371/journal.pone.0202801)

S1 Fig.

Flow diagram of patient selection with angiotensin-converting enzyme inhibitors as referent group


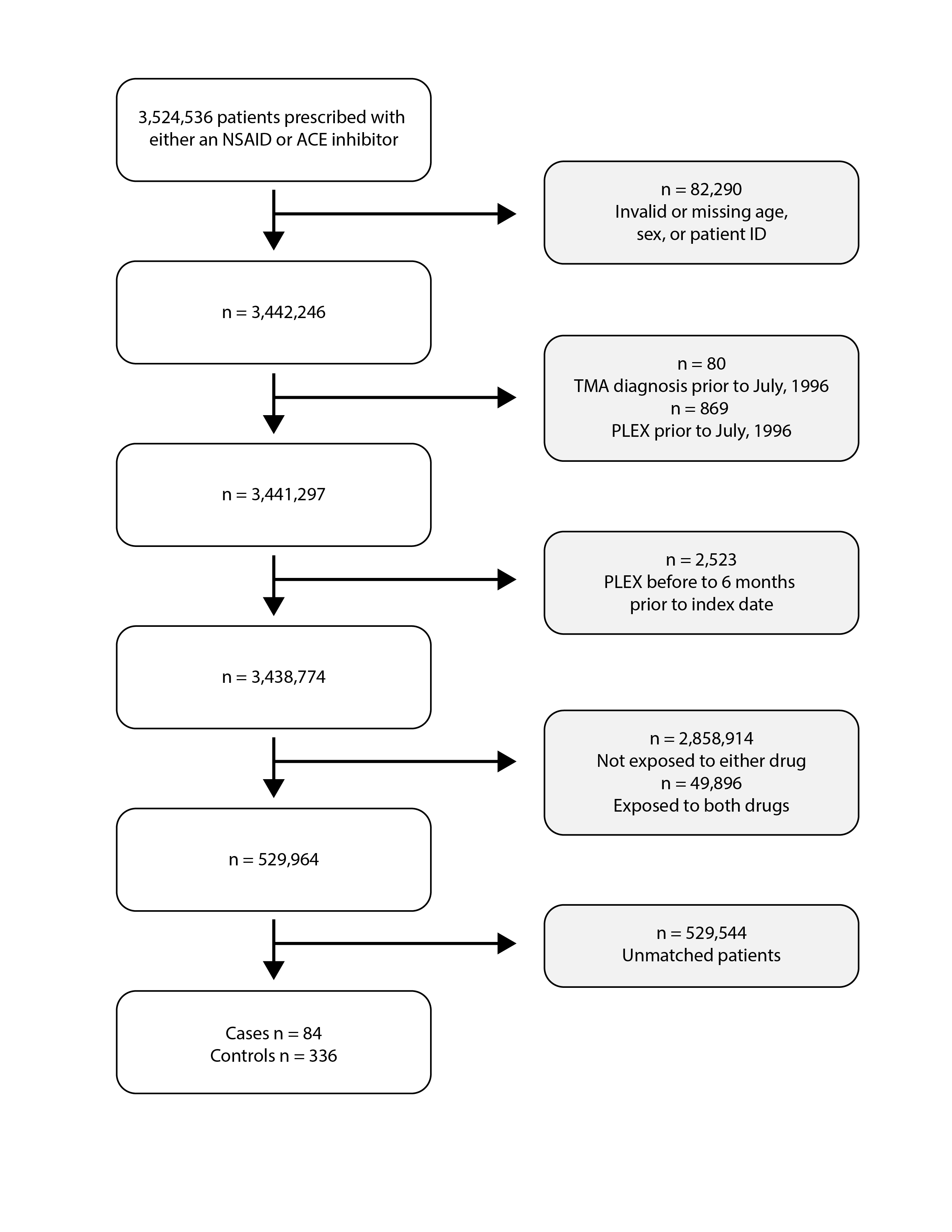

Supplement: S1 Fig — (DOCX) [file pone.0202801.s001.docx]
