# Supplementary material for: A matched case-control study to assess the association between non-steroidal anti-inflammatory drug use and thrombotic microangiopathy
Source: PLoS One. 2018 Aug 24;13(8):e0202801. doi: 10.1371/journal.pone.0202801 (PMC6108507; doi:10.1371/journal.pone.0202801)
Supplement: S1 Table — (DOCX) [file pone.0202801.s002.docx]

S1 table

**Case report appraisal**

| **Case report evaluation for NSAID associated TMA** | | | | |
| --- | --- | --- | --- | --- |
| **NSAID** | **Citation** | **Year published** | **Patient sex and age in years** | **Level of evidence^1^** |
| Diclofenac | Claros González I, Baños Gallardo M, Casal Alvarez F, Argüelles Toraño M. Systemic thrombotic microangiopathy secondary to diclofenac. *Med Clínica*. 1989;92(10):396. | 1989 | Male, middle aged | 5 |
| Ibuprofen | Catizone L, Santoro A, Scialfa G, Cagnoli L, Fabbri L. Thrombotic thrombocytopenic purpura due to administration of Ibuprofen. *Minerva Nefrol*. 1974;21(6):439-444. | 1974 | Female, 55 | 5 |
| Ibuprofen | Schoenmaker NJ, Weening JJ, Krediet RT. Ibuprofen-induced HUS. *Clin Nephrol*. 2007;68(3):177-178. | 2007 | Female, 44 | 2 |
| Ibuprofen | Oregel KZ, Ramdial J, Glück S. Nonsteroidal Anti-inflammatory Drug Induced Thrombotic Thrombocytopenic Purpura. *Clin Med Insights*. 2013;6:19-22. doi:10.4137/CMBD.S12843. | 2013 | Male, 21 | 2 |
| Ibuprofen | Benmoussa J, Chevenon M, Nandi M, Forlenza TJ, Nfonoyim J. Ibuprofen-induced thrombotic thrombocytopenic purpura. *Am J Emerg Med*. 2016;34(5):942.e5-e7. doi:10.1016/j.ajem.2015.10.044. | 2016 | Male, 37 | 2 |
| Ketorolac Tromethamine | Randi ML, Tison T, Luzzatto G, Girolami A. Haemolytic uraemic syndrome during treatment with ketorolac trometamol. *BMJ*. 1993;306(6871):186. | 1993 | Female, 58 | 2 |
| Naproxen | Trice JM, Pinals RS, Plitman GI. Thrombotic thrombocytopenic purpura during penicillamine therapy in rheumatoid arthritis. *Arch Intern Med*. 1983;143(7):1487-1488. doi:10.1001/archinte.1983.00350070215039. | 1983 | Male, 64 | 3 |
| Pranoprofen | Okura H, Hino M, Nishiki S, et al. Recurrent hemolytic uremic syndrome induced by pranoprofen. *Rinsho Ketsueki*. 1999;40(8):663-666. | 1999 | Female, 25 | 2 |

^1^ Case reports are given a level from 1 to 5 depending on how many causal criteria the case fulfills; 1 = definite evidence of a causal relationship, 2 = probable, 3 = possible, 4 = unlikely, 5 = unsuitable for review. A more detailed explanation is provided in Al-Nouri et al.
