# Supplementary material for: A matched case-control study to assess the association between non-steroidal anti-inflammatory drug use and thrombotic microangiopathy
Source: PLoS One. 2018 Aug 24;13(8):e0202801. doi: 10.1371/journal.pone.0202801 (PMC6108507; doi:10.1371/journal.pone.0202801)
Supplement: S3 Table — (DOCX) [file pone.0202801.s004.docx]

S3 table

| Coding definitions for cohort build and baseline characteristics | | |
| --- | --- | --- |
| Variable | Database | Codes |
| Cohort Selection | | |
| TMA | CIHI-DAD | ICD-9: “4466”  ICD-10: “M311” |
| Plasma Exchange | OHIP | “G272”, “G277”, “G278”, “G290” |
| NSAIDs | ODB | CELECOXIB, DICLOFENAC, DICLOFENAC SODIUM, DICLOFENAC SODIUM & MISOPROSTOL, DIFLUNISAL, ETODOLAC, FENOPROFEN CALCIUM, FLOCTAFENINE, FLURBIPROFEN, IBUPROFEN, INDOMETHACIN, KETOPROFEN, KETOROLAC TROMETHAMINE, MEFENAMIC ACID, MELOXICAM, NABUMETONE, NAPROXEN, OXAPROZIN, PIROXICAM, ROFECOXIB, SULINDAC, TIAPROFENIC ACID, TOLMETIN SODIUM, VALDECOXIB |
| Acetaminophen | ODB | ACETAMINOPHEN & CODEINE PHOSPHATE, ACETAMINOPHEN & CAFFEINE & CODEINE PHOSPHATE |
| Dilaudid | ODB | HYDROMORPHONE, HYDROMORPHONE HCL |
| ACE inhibitors | ODB | BENAZEPRIL CHLOROHYDRATE, BENAZEPRIL HCL, CAPTOPRIL, CILAZAPRIL, ENALAPRIL SODIUM, FOSINOPRIL, FOSINOPRIL SODIUM, LISINOPRIL, PERINDOPRIL TERT.BUTYLAMINE, QUINAPRIL, RAMIPRIL, TRANDOLAPRIL |
| Baseline comorbidities | | |
| Cancers | CIHI-DAD  OHIP | ICD9 (CIHI-DAD): "150", "154", "155", "157", "162", "174", "175", "185", "203", "204", "205", "206", "207", "208", "2303", "2304", "2307", "2330", "2312", "2334"  IDC10 (CIHI-DAD): "971", "980", "982", "984", "985", "986", "987", "988", "989", "990", "991", "993", "C15", "C18", "C19", "C20", "C22", "C25", "C34", "C50", "C56", "C61", "C82", "C83", "C85", "C91", "C92", "C93", "C94", "C95", "D00", "D05", "D010", "D011", "D012", "D022", "D075"  OHIP DX: "203", "204", "205", "206", "207", "208", "150", "154", "155", "157", "162", "174", "175", "183", "185" |
| Kidney transplant | CORR  OHIP | CORR:  RECIPIENT_TREATMENT dataset  [Treatment_Code]: 171  [Treatment_Date]  [Transplanted_Organ_Type_Code][1-3]: "10", "11", "12", "18", "19"  CCP: "6759"  CCI: "1PC85"  OHIP feecode: "S435", "S434" |
| Rheumatoid arthritis |  | ICD9: "714"  ICD10: "M05", "M06"  OHIP Dx: "714" |
| Osteoarthritis | CIHI-DAD | ICD9: "715"  ICD10: "M15", "M150", "M151", "M152", "M153", "M154", "M158", "M159" |
| Malignant hypertension | CIHI-DAD | ICD9: "4010"  ICD10: "I101" |
| Systemic lupus erythematosus | CIHI-DAD | ICD9: "7100"  ICD10: "M320", "M321", "M328", "M329" |
| HIV | CIHI-DAD  OHIP | ICD9 (CIHI-DAD): "042", "043", "044", "176"  ICD10 (CIHI-DAD): "B24", "Z21", "C46"  OHIP DX: "042", "043", "044" |
| Sepsis | CIHI-DAD | ICD9: "0031", "0362", "0380", "0381", "0382", "0383", "03840", "038.41", "03842", "03843", "03844", "03849", "0388", "0389"  ICD10: "A40", "A41" |

TMA: Thrombotic microangiopathy, NSAIDs: non-steroidal anti-inflammatory drugs, ICD: International Classification of Diseases, CIHI-DAD: Canadian Institute for Health Information – Discharge Abstract Database, OHIP: Ontario Health Insurance Plan, CORR: Canadian Organ Replacement Register, CCI: Canadian Classification of Health Interventions, CCP: Canadian Classification of Diagnostic, Therapeutic and Surgical Procedures, ACE: angiotensin-converting enzyme, HIV: Human Immunodeficiency Virus
