# Supplementary material for: A matched case-control study to assess the association between non-steroidal anti-inflammatory drug use and thrombotic microangiopathy
Source: PLoS One. 2018 Aug 24;13(8):e0202801. doi: 10.1371/journal.pone.0202801 (PMC6108507; doi:10.1371/journal.pone.0202801)
Supplement: S4 Table — (DOCX) [file pone.0202801.s005.docx]

S4 table

**Baseline characteristics for patients prescribed NSAIDs^1^ or ACE-inhibitors^1^ with and without thrombotic microangiopathy (cases and controls, respectively)**

|  | **Controls**  **(n=336)** | **Cases**  **(n=84)** | **P-value^4^** |  |
| --- | --- | --- | --- | --- |
| **Demographics** | | | |  |
| Age, no. (%) | | | |  |
| Median (IQR) | 74 (68-81) | 74 (67-82) |  |  |
| Mean ± SD | 73 ± 10.4 | 73 ± 11.13 | 0.22 |  |
| ≤ 17 | 21 (6.3%) | 0 | 0.41 |  |
| 18 - 44 |  | 0 |  |  |
| 35 - 44 |  | ≤5 |  |  |
| 16 - 54 |  | ≤5 |  |  |
| 55 - 64 | 20 (6%) | 7 (8.3%) |  |  |
| 65 - 74 | 140 (41.7%) | 37 (44%) |  |  |
| 75 - 84 | 112 (33.3%) | 22 (26.2%) |  |  |
| ≥ 85 | 43 (12.8%) | 13 (15.5%) |  |  |
| Female, no. (%) | 200 (59.5%) | 50 (59.5%) | 1.0 |  |
| Rural location, no. (%)^2^ | 56 (16.7%) | 14 (16.7%) | 1.0 |  |
| Socioeconomic status, no. (%)^3^ | | | |  |
| Quintile 1 | 72 (21.4%) | 18 (21.4%) | 1.0 |  |
| Quintile 2 | 56 (16.7%) | 14 (16.7%) | 1.0 |  |
| Quintile 3 | 56 (16.7%) | 14 (16.7%) | 1.0 |  |
| Quintile 4 | 72 (21.4%) | 18 (21.4%) | 1.0 |  |
| Quintile 5 | 80 (23.8%) | 20 (23.8%) | 1.0 |  |
| Primary care physician visits, no. (%) | | | |  |
| Median (IQR) | 8 (5-13) | 12 (7-19) |  |  |
| Mean ± SD | 11 ± 11.26 | 16 ± 14.23 | <0.01 |  |
| 0 | 16 (4.8%) | ≤5 | <0.01 |  |
| 1 - 2 | 34 (10.1%) | 8 (9.5%) |  |  |
| 3 - 4 | 54 (16.1%) | ≤5 |  |  |
| 5 - 6 | 54 (16.1%) | 6 (7.1%) | <0.01 |  |
| 7 - 8 | 37 (11%) | 9 (10.7%) |  |  |
| 9 - 10 | 38 (11.3%) | 9 (10.7%) |  |  |
| ≥ 11 | 103 (30.7%) | 47 (56%) |  |  |
| **Comorbidities, no. (%)** | | | |  |
| John Hopkins ADG Score, no. (%) | | | |  |
| Median (IQR) | 11 (8-14) | 13.5 (11-16) |  |  |
| Mean ± SD | 11 ± 4.19 | 13 ± 3.68 | <0.01 |  |
| ≤ 9 | 122 (36.3%) | 12 (14.3%) | <0.01 |  |
| 10 - 12 | 92 (27.4%) | 23 (27.4%) |  |  |
| 13 - 15 | 62 (18.5%) | 22 (26.2%) |  |  |
| ≥ 16 | 60 (17.9%) | 27 (32.1%) |  |  |
| Malignant hypertension | ≤5 | ≤5 | - |  |
| Systemic lupus erythematosus | ≤5 | ≤5 | - |  |
| Cancer | 32 (9.5%) | 12 (14.3%) | <0.01 |  |
| Renal transplant | ≤5 | ≤5 | - |  |
| Osteoarthritis | 11 (3.3%) | ≤5 | <0.01 |  |
| Rheumatoid arthritis | 18 (5.4%) | 9 (10.7%) | <0.01 |  |
| HIV^1^ | ≤5 | ≤5 | 1.0 |  |
| Sepsis | 8 (2.4%) | ≤5 | 1.0 |  |
| **Medications, no. (%)** | | | |  |
| Quinine | ≤5 | ≤5 | - |  |
| Quetiapine | ≤5 | ≤5 | - |  |
| Tacrolimus | ≤5 | ≤5 | - |  |
| Sirolimus | ≤5 | ≤5 | - |  |
| Cyclosporine | ≤5 | ≤5 | - |  |
| Clopidogrel | ≤5 | ≤5 | - |  |
| Ticlopidine | ≤5 | ≤5 | - |  |

^1^no.: Number, IQR: interquartile range, SD: Standardized difference, NSAIDs: non-steroidal anti-inflammatory drugs, ACE: angiotensin-converting enzyme, HIV: Human immunodeficiency virus

^2^Rural residence is defined as population < 10,000

^3^Quntiles are ranked from lowest to highest (i.e. Quintile 1 = lowest, Quintile 5 = highest)

^4^P-values are calculated using generalized estimating equations
