# Supplementary material for: A matched case-control study to assess the association between non-steroidal anti-inflammatory drug use and thrombotic microangiopathy
Source: PLoS One. 2018 Aug 24;13(8):e0202801. doi: 10.1371/journal.pone.0202801 (PMC6108507; doi:10.1371/journal.pone.0202801)
Supplement: S5 Table — Odds ratios derived from a conditional logistic regression model. (DOCX) [file pone.0202801.s006.docx]

S5 table

**The association between NSAID use and thrombotic microangiopathy, with ACE inhibitors as a reference group. Odds ratios derived from a conditional logistic regression model.**

|  | **Cases** | **Controls** | **Odds Ratio (95% confidence interval)** | |
| --- | --- | --- | --- | --- |
|  |  |  | **Unadjusted** | **Adjusted^1^** |
| ACE inhibitors^2^ | 66 | 253 | 1.0 (referent) | 1.0 (referent) |
| NSAIDs^2^ | 18 | 83 | 0.82 (0.45-1.49) | 0.72 (0.38-1.37) |

1Adjusted analysis included the following variables: cancer, osteoarthritis, rheumatoid arthritis, John Hopkin’s ADG score and primary care physician visits

^2^NSAIDs: Non-steroidal anti-inflammatory drugs, ACE: angiotensin-converting enzyme
